# *KIF1A*-related autosomal dominant spastic paraplegias (SPG30) in Russian families

**DOI:** 10.1186/s12883-020-01872-4

**Published:** 2020-08-03

**Authors:** G. E. Rudenskaya, V. A. Kadnikova, O. P. Ryzhkova, L. A. Bessonova, E. L. Dadali, D. S. Guseva, T. V. Markova, D. N. Khmelkova, A. V. Polyakov

**Affiliations:** 1 Federal State Budgetary Scientific Institution “Research Centre for Medical Genetics” (RCMG), Moscow, Russia; 2 “Genomed” Ltd, Moscow, Russia

**Keywords:** Autosomal dominant spastic paraplegia type 30 (SPG30), *KIF1A* gene, Pathogenic variants, Gene panel, Massive parallel sequencing, Pure phenotype, Additional features, Phenotypic variability

## Abstract

**Background:**

Spastic paraplegia type 30 (SPG30) caused by *KIF1A* mutations was first reported in 2011 and was initially considered a very rare autosomal recessive (AR) form. In the last years, thanks to the development of massive parallel sequencing, SPG30 proved to be a rather common autosomal dominant (AD) form of familial or sporadic spastic paraplegia (SPG),, with a wide range of phenotypes: pure and complicated. The aim of our study is to detect AD SPG30 cases and to examine their molecular and clinical characteristics for the first time in the Russian population.

**Methods:**

Clinical, genealogical and molecular methods were used. Molecular methods included massive parallel sequencing (MPS) of custom panel ‘spastic paraplegias’ with 62 target genes complemented by familial Sanger sequencing. One case was detected by the whole -exome sequencing.

**Results:**

AD SPG30 was detected in 10 unrelated families, making it the 3rd (8.4%) most common SPG form in the cohort of 118 families. No AR SPG30 cases were detected. In total, 9 heterozygous *KIF1A* mutations were detected, with 4 novel and 5 known mutations. All the mutations were located within *KIF1A* motor domain. Six cases had pure phenotypes, of which 5 were familial, where 2 familial cases demonstrated incomplete penetrance, early onset and slow relatively benign SPG course. All 4 complicated cases were caused by novel mutations without familial history. The phenotypes varied from severe in two patients (e.g. lack of walking, pronounced mental retardation) to relatively mild non-disabling symptoms in two others.

**Conclusion:**

AD SPG30 is one of the most common forms of SPG in Russia, the disorder has pronounced clinical variability while pure familial cases represent a significant part.

## Background

The heterogeneous group of hereditary spastic paraplegias (HSP) includes over 80 genetic types that are designated SPG (Spastic Paraple*g*ia), numbered in the order of their discovery. The vast majority of genes are identified, few remain only mapped. A lot of SPGs were recognized recently thanks to methods of massive parallel sequencing (MPS). Apart from the identification of new forms, MPS adds knowledge about earlier discovered SPG, refines their phenotypes, epidemiology and classification.

One of SPG genes is *KIF1A* (Kinesin Family1A) – neuron-specific motor protein involved in intracellular microtubuleaxonal transport of a variety of vesicles along microtubules that contribute to pre- and post-synaptic assembly, autophagic processes and neuron survival. HSP-related kinesin-3 mutants are characterized mainly as loss-of-function resulting in deficits in motility, regulation, and cargo binding. Gain-of-function mutants are also seen, and are characterized by increased microtubule-on rates and hypermotility. Both sets of mutations ultimately result in misdelivery of critical cargos within the neuron. This likely leads to deleterious cell biological cascades that likely underlie or contribute to HSP clinical pathology and ultimately, symptomology [1]. The vast majority оf pathogenic mutations are located within the gene’s motor domain which contains amino acids from 5 to 354 of the total 1690. *KIF1A* mutations cause different HSP phenotypes and demonstrate different modes of inheritance. In 2006 autosomal recessive (AR) HSP with mild ataxia and sensory neuropathy was mapped to the locus 2q37.3 containing *KIF1A* and assigned to SPG30 (OMIM#610357) [2]. The AR SPG30 were since identified in a few other mostly consanguineous families [3–5], making AR SPG30 a very rare form of HSP. While, cases of mental retardation (MR) with neurological symptoms (mostly progressive spastic paraparesis) caused by autosomal dominant (AD) *KIF1A* mutations acquired de novo were reported more and more often starting from 2011. They were named MR type 9 (MR9, OMIM#614255) [6–9] or complicated AD SPG30 [10–12]. In addition, cases of uncomplicated (‘pure’) AD SPG30, mostly familial, have been increasingly diagnosed [10, 13, 14] (elaborated in Discussion). Thus, in some ethnical groups AD SPG30 is among the most common forms of SPG [15, 16]. Surprisingly, AD SPG30 (‘pure’ phenotypes in particular) is not included in the OMIM, also SPG30 is represented only by a very rare AR form that may mislead geneticists and neurologists.

To summarize, within a short period of time the view of SPG30 as of a rare AR SPG has been changed dramatically. SPG30 appeared to be more frequent pathology, with prevailing AD inheritance and with pronounced clinical variability, ranging from uncomplicated SPG to severe forms when spastic paraplegia might not always be the leading symptom.

The article presents a first representative study of AD SPG30 in the Russian population, an overview of cases that have been detected by MPS with additional molecular methods.

## Methods

### Patients

Ten АD SPG30 families were selected based on clinical, genealogical, and molecular diagnostics. HSP was clinically diagnosed at the Research and Counseling Department of the Research Centre for Medical Genetics (RCMG) and SPG30 diagnosis was confirmed at the molecular level in the DNA diagnostics laboratory in 2017–2019. In 1 of the total 10 cases DNA testing was performed in ‘Genomed’ Laboratory.

The ten families come from different regions of Russia. Six families are ethnically Russians, one Russian Serbs family, two families are ethnically Tatars, and one family is Dargin (a Dagestan ethnicity). Index cases (IC) are 5 males and 5 females, age at analysis 5–59 years, manifestation mostly 1–2-nd decades; 5 affected relatives were examined clinically. Information about family members unavailable for personal clinical examination was received from their relatives and/or medical papers.

### Molecular methods

Genomic DNA was extracted from the whole venous blood with Wizard® Genomic DNA Purification Kit (Promega, USA) following the manufacturer’s protocol.

For the current research the “Spastic Paraplegia” Sequencing Panel of target genes was used. The custom MPS panel includes 62 genes (56 SPG genes and 6 genes of spastic ataxias): *GJC2, AP4B1, AMPD2, IBA57, ALDH18A1, ZFYVE27, NT5C2, ENTPD1, MTPAP, CAPN1, BSCL2, KLC2, KIF5A, C12orf65, MARS, VAMP1, B4GALNT1, SPG20, SACS, ATL1, ZFYVE26, DDHD1, TECPR2, AP4S1, NIPA1, SPG11, SPG21, AP4E1, USP8, SPG7, FA2H, ARL6IP1, KIF1C, AFG3L2, RTN2, PNPLA6, C19orf12, CPT1C, MAG, HSPD1, KIF1A* (NM_004321.6)*, REEP1, PGAP1, MARS2, SPAST, SLC33A1, TFG, WDR48, CYP2U1, ARSI, ZFR, REEP2, AP5Z1, AP4M1, CYP7B1, KIAA0196, ERLIN2, VPS37A, DDHD2, GBA2, L1CAM, PLP1* and *SLC16A2*.

MPS of patient’s DNA was performed by Ion S5 next-generation sequencer (Thermo Fisher Scientific, USA) with an Ion AmpliSeq™ Library Kit 2.0 according to the manufacturer’s protocol.

MPS was complemented by familial Sanger sequencing and MLPA (Multiplex Ligation-dependent Probe Amplification) (selectively). Few cases with unclear clinical picture were diagnosed by whole-exome sequencing (WES) that unraveled *KIF1A* mutations. All detected variants were reconfirmed via Sanger sequencing.

Sequencing data was processed using a standard algorithm from Thermo Fisher Scientific (Torrent Suite™) and Gene-Talk software (www.gene-talk.de/contact; Gene Talk GmbH, Germany). Sequenced fragments were visualized via the Integrative Genomics Viewer (IGV) software (© 2013–2018 Broad Institute, and the Regents of the University of California, USA).

The Genome Aggregation Database (gnomAD v 2.1.1) was used to determine the incidence rate of newly discovered variants.

The following online prediction programs were utilized to determine pathogenicity in silico: Mutation Taster (http://www.mutationtaster.org/), UMD-predictor (http://umd-predictor.eu/); SIFT/Provean (http://provean.jcvi.org/index.php); PolyPhen-2 (http://genetics.bwh.harvard.edu/pph2/index.shtml); and Human Splicing Finder (http://www.umd.be/HSF/).

Guidelines for interpretation of MPS data [17, 18] were used to define the clinical significance of newly discovered variants.

According to MPS findings and bioinformatic analysis DNA testing of relatives was performed: verification of mutation origin (in all sporadic cases, in particular) and/or search of mutations in affected relatives and relatives at risk. Blood samples of persons not examined personally were transmitted.

## Results

By now, our cohort of molecularly diagnosed SPG encloses 118 unrelated families with 21 genetic forms. SPG30 is the 3rd (8.4%) most common form of HSP after prevalent SPG4 (60 cases) and SPG3 (15 cases), and in the subgroup of HSP forms with AD heredity SPG30 amounts for 10.3%. No AR *KIF1A-*related cases were detected in our cohort. Main genetic and clinical characteristics of 10 families diagnosed with SPG30 are presented in Table 1.

**Table 1:** Genetic and clinical features of AD SPG30 in 10 Russian families

Family, оrigin	*KIF1A* mutation	Ex-on	Familial /sporadic			Personally examined patients				Other patients
				Family member	Age, y	Onset age, y	Pyramidal signs	Gate impairment	Additional signs	
30–1 Russian	с.22G > A (Val18Met)	2	Familial	IC, F	59	1	++	+++ Aided since 55 y	Incontinence	Daughter-1: early onset, ambulant till death in 35 y daughter-2 & grandson – asymptomatic
30–2 Russian	с.37С > T (p.Arg13Cys) de novo	2	Sporadic	IC, M	7	Congenital	++	+++ Unaided	MR, stereotypias, dysarthria, enuresis, obesity	–
30–3 Russian	с.206C > T (p.Ser69Leu) de novo	3	Sporadic	IC, F	33	2	++	++ Unaided	–	–
30–4 Dargin	с.206C > T (p.Ser69Leu)	3	Familial	IC, F	15	3	+++	++ Unaided	–	Grandfather & 3 uncles – early onset, ambulant
				Father	45	Childhood	++	+ Unaided	–	
30–5 Tatar	с.220 T > C (p.Tyr74His)	4	Familial	IC, М	5	4–5	++	+ Unaided		Uncle-1: early onset, ambulant; uncle-2 asymptomatic
				Father	36	SS	+/−	+/−	–	
30–6 Russian	с.499C > T (p.Arg167Cys)	5	Familial	IC М	17	16, SS - 9		++	–	–
				Father	43	Childhood		++	–	
				Grand-father	74	After 40	++	Aided since 55 y till death	–	
				Aunt-1	50	45	++	++	–	
				Aunt-2	47	18–20	++	++	–	
30–**7** Tatar	c.607A > G (p.Arg203Gln) de novo	6	Sporadic	IC, М	12	Congenital	++	No walking	MR, microcephaly, microsomia, МRI N	–
30–8 Russian	с.761G > A (p.Arg254Gln)	7	Familial	IC, F	5	2–3	++	+ Unaided	–	Grandfather: impaired gate since youth, feet deformity
				Mother	33	Congenital	++	+++ Unaided	–	
30–9 Russian- Serb	с.798 + 1G > T de novo	8	Sporadic	IC, М	19	13 SS since 7 y	++	++ Unaided	Mild cognitive deficiency; ASD?	–
30–10 Russian	c.917A > G (p.Tyr306Cys) de novo	11	Sporadic	IC, F	5	Congenital	+/−	Mildly atactic, no spasticity Unaided	Mild mental & speech delay, cataract MRI: hypogenesia of corpus callosum	–

*IC* index case, *M* male, *F* female, *SS* subclinical signs, *MR* mental retardation, *MRI* magnetic resonance imaging, *ASD* autistic spectrum disorder, *N* normal

### Molecular characteristics

In 10 families 9 different *KIF1A* mutations were detected. All mutations were in exons 2–11 encoding *KIF1A* motor domain. Five mutations were reported earlier, where the mutation с.206C > T (p.Ser69Leu) that was found in a Russian (30–1) and in a Dargin (30–4) families has been already reported in several families of a different origin [10, 13, 16, 19, 20].

The pathogenicity of 4 novel mutations is shown in Table 2. Familial DNA testing confirmed mutations in 7 affected relatives and in 4 asymptomatic/subclinical cases (2 in 30–1 and 2 in 30–5). In 21 unaffected participants mutations were not found. In families 30–2, 30–3, 30–7, 30–9 and 30–10 the absence of mutations in both parents confirmed de novo origin of SPG30.

**Table 2:** Pathogenicity of the *KIF1A* novel variants

Family	Variant	Exon	Pathogenicity	Criteria
30–2	c.37C > T p.Arg13Cys	2	Likely pathogenic	PS2, PM1, PM2, PP3
30–7	c.607A > G p.Arg203Gln	6	Likely pathogenic	PS2, PM1, PM2, PP3
30–9	c.798 + 1G > T	8	Pathogenic	PVS1, PS2, PM1, PM2, PP3
30–10	c.917A > G p.Tyr306Cys	11	Likely pathogenic	PS2, PM1, PM2, PP3

### Genealogical and clinical characteristics

**Fig. 1:**
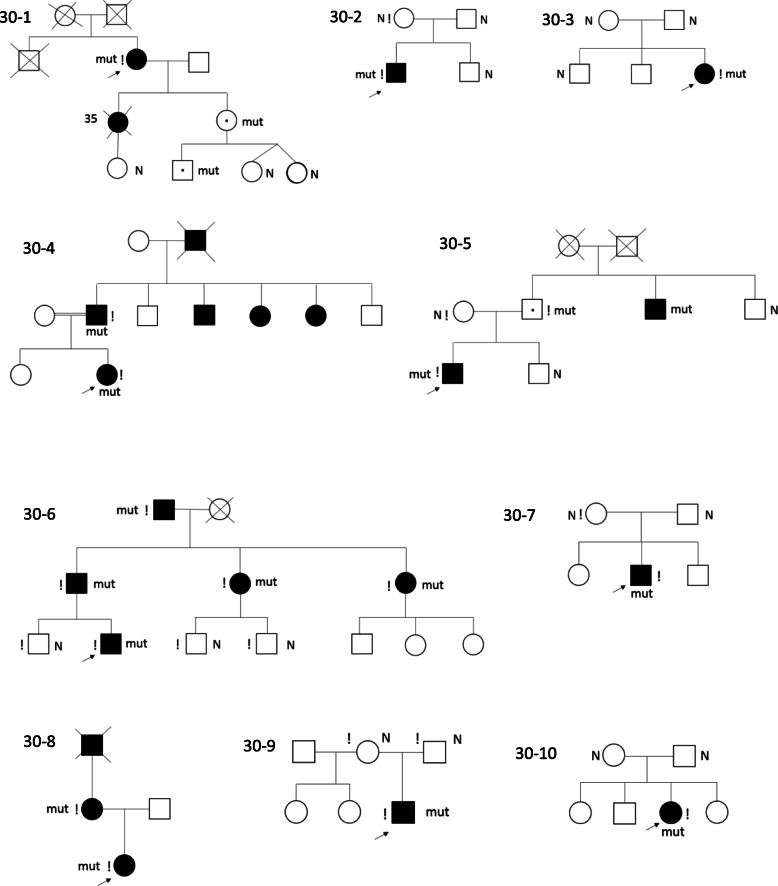
Pedigrees of SPG30 families. Legend: – affected person; − proband;! – examined patient; mut – pathogenic variant; – deceased; – asymptomatic carrier of pathogenic variant

Russian cohort demonstrates a wide spectrum of genealogical and phenotypic variants of SPG30. Pedigrees are shown in Fig. 1. Five cases are familial with AD inheritance in families 30–1, 30–4, 30–5, 30–6, 30–8. Two familial AD cases 30–1 and 30–5 have “missing cases” in family history, i.e. asymptomatic persons with mutations, pointing to incomplete penetrance (or preclinical stage in a child in 30–1). In case 30–5 the incomplete penetrance was evident from the pedigree (e.g. affected IC and uncle but non-affected father) and confirmed by familial DNA testing. In 30–1 missing cases were found by DNA test of clinically unaffected relatives. Five cases are sporadic and caused by mutations de novo. Interestingly, all 5 familial cases were caused by known mutations, whereas 4 out of 5 mutations acquired de novo were novel, and only one sporadic case 30–3 was caused by a well-known mutation p.Ser69Leu.

Six cases, 30–1, 30–3 – 30-6 and 30–8, demonstrated typical uncomplicated HSP in all affected members with predominantly early onset and relatively benign slow course. All patients in these families were ambulatory and were able to walk without support even in cases of pronounced gait disturbances; only IC in 30–1 and grandfather in 30–6 used stick or crutches at the age over 60 years. Spastic paraparesis had typical features: leg hyperreflexia, variable set of flexor and/or extensor “pathological feet signs”, feet clonus, spastic gait of differing degree (though high leg tone in lying position was seen only in 3 patients). ‘Friedreich’s’ feet deformation was present in 7 patients from these 6 families as in children it may develop later. Arm lesion was evident only by brisk reflexes in 4 patients. Bladder dysfunction was a rare sign: persistent incontinence from the age of 40 years in 30–1 IC, and mild dysfunction in 30–4 IC. Brain magnetic resonance imaging (MRI) was performed only in 3 out of 6 ‘pure’ cases (IC and affected daughter in 30–1; IC in 30–5; IC and affected mother in 30–8) and was within the norm. Family 30–6 which we followed up for many years was the only one with interfamilial differences of clinical symptoms onset age: ranging from childhood up to the 5th decade, yet there were no significant differences in the severity. Another example of interfamilial differences of HSP course is in 30–4: all 5 patients had childhood onset, but 15-year old IC had more pronounced symptoms compared to her affected elder relatives in similar age. On the contrary, in the family 30–8 the clinical state of IC was better than of her mother in childhood. Convincing evidence of interfamilial variability is the presence of incomplete penetrance in families 30–1 and 30–5. Overall 6 ‘pure’ cases were clinically alike and relatively benign.

The opposite end of the HSP clinical spectrum is presented by two sporadic severe ‘complicated’ cases, 30–2 and particularly 30–7, described in more detail below.

The patient in sporadic case 30–2 had normal pre- and neonatal periods. He walked on toes since the age of independent walking at 2 years and 9 months; after surgery on Achilles tendon at 4 years his gait improved but when he became 6 years old spasticity increased again. At the examination time, being 7.5 years old he was able to walk without aid, but slowly, unsteadily, with fallings, gait is spastic with leg recurvation and elements of paretic. Speech and mental delay in infancy were mild, but at 3–5 years retardation progressed. At 7.5 years the boy has poor dysarthric speech (separate words, few short phrases). He communicates emotionally and understands simple speech; elementary self-care skills are partially developed. There is no sensible play, stereotypic movements are evident. Brain MRI was within the norm. Astigmatism was the only vision symptom. Since 6 years age he was obese, due to the feeding behavior. Phenotype at the examination: body mass 40 kg (> 97 centile), height 130 cm (> 95 centile), body mass index 100 centile, head circumference 54 cm, facial features prominent incisors, poor dermatoglyphics; tendon reflexes are brisk (in legs more than in arms), gait was slow, spastic with elements paretic and leg recurvation; mental retardation (MR), quiet behavior, stereotypies.

The 12-year-old boy in de novo case 30–7 has the most severe phenotype in the cohort. He was born by emergency caesarian section with the birth mass of 3700 g and 7/8 Apgar score. In the neonatal period his state was of moderate severity. Head control appeared timely but after that, the overall motor development has been hampered and spastic tetraparesis with predominant lower paraparesis has developed. At the age of 10 months cerebral palsy was diagnosed. Thank to rehabilitation treatment, the boy was able to seat at 1.5 years. He tried supported walking, but multiple contractures and feet deformities developed quickly, and he remained non-ambulant. MR is severe: he recognizes family members, but has no sensible contact, the speech represents senseless sounds only, self-care skills are absent, etc.; stereotypic movements and laughter are prominent. There is no epilepsy or electroencephalographic (EEG) epileptiform activity in this patient. Brain MRI in 2 years was normal. Phenotype: the boy does not look according to his age, he has severe height and weight deficiency: 131 cm (< 3 cent); 19 kg (< 50 cent); body mass index 11,07 (< 3 cent); microcephaly 40 cm (< 2 SD); dysmorphic features: large incisors and broad alveolar processes; skeletal signs: kyphoscoliosis, feet combined deformity, contraсtures of hip, knee and ankle joints S > D; moderate arm and severe leg spastic paraparesis, arm reflexes are brisk, knee and Achilles reflexes are not triggered due to contractures, Babinski sign S, stereotypic movements, severe MR (see above).

Cases 30–2 and 30–7 were in line with the diagnosis of MR type 9 with spastic paraparesis (or severe complicated AD SPG30) that was confirmed by MPS followed by familial Sanger sequencing.

In case 30–9 the 19-year-old youth is the only child in a mixed Russian-Serb family. Since early childhood, he had a moderate awkwardness, e.g. he ran clumsily on toes (but walked on full soles). Mild cerebral palsy was suspected. At the age of 13 years the parents noticed gait worsening that was not felt by the boy himself. He studied poorly in an ordinary school in spite of the additional tutoring, and now he studies in a college with simplified program. His interests are limited and not age-matching, he has no friends or girl-friends; a general behavior is adequate. The mother denied cognitive and behavior problems in the son but the father acknowledged them; parents refused the patient’s psychological/neuropsychological examination. Brain MRI and EEG are normal. There are no dysmorphic features. Neurological examination detected spastic paraparesis without feet deformation, with normal parameters including leg tone in lying position, brisk polykinetic wide-zone knee reflexes, feet clonus, all extension and Rossolimo feet signs; the patient’s gait was moderately spastic and unaided; urinary functions, abdominal reflexes, sensitivity and coordination were all normal. At the examination the youth behaved quietly, did not communicate actively, answered questions shortly but adequately; there was an impression of autistic-like features. Family 30–9 case may be considered intermediate between ‘pure’ SPG30 and complicated form with the evident MR, though autistic spectrum disorder (ASD) was possible.

The case of family 30–10 is the only case for which DNA tests (WES followed by familial Sanger sequencing) were performed in another medical institution, ‘Genomed’ laboratory, subsequently this family has been examined clinically in RCMG. The phenotype observed in the 5-year-old girl (the only affected child out of 5 siblings) stands apart as, ataxia symptoms were predominant while spastic paraplegia was not prominent. Pre- and perinatal periods were without complications. Head control and sitting developed timely but she was able to walk independently only in 2 years and 4 months, and then ataxia became evident. With aging the degree of ataxia decreased, though clumsiness without spasticity persisted. Self-care skills formed timely. Phrasal speech with moderate dyslalia developed in 3 years. The girl behaved and contacted adequately. Brain MRI detected mild hypogenesia of corpus callosum; cerebellum was normal. Extraneural signs included congenital bilateral punctual cataract and macular hypoplasia with preserved vision in everyday life. Physical examination showed strabismus OS, normal muscle tone, normal upper limbs and brisk lower limbs reflexes without pathological pyramidal signs, intention in finger-to-nose probe, instability in Romberg’s position, awkwardness in walking and running were and the, absence of ataxia in the gait. MPS-panel “neurodegenerations” detected no other potentially pathogenic indications apart from *KIF1A* novel mutation proven to appear de novo. Three years later, according to the information from the mother and medical documents, the dynamics is positive, the girl can read and write, she began to study at an ordinary school (though has difficulties with math), mild dyslalia and awkwardness in walking and running persist. The new neurological signs that have developed include nystagmus, and brisk reflexes in upper limbs, other signs were the same. MRI was not repeated. Ophthalmological surgery (bilateral cataract resection and artificial lens implantation) was performed. This relatively mild neurological disorder with partial improvement does not match to the typical SPG (pyramidal signs are presented only by brisk reflexes). Yet the case have been included in our patients cohort as the thinning of corpus callosum is characteristic feature of several SPG and was reported in AD SPG30, besides ataxia was also reported in several cases though it is not the main feature. The cataract is the novel symptom of SPG30.

One additional occurrence of *KIF1A* mutation was not included in the group of selected families. In a Kirghiz non-consanguineous family from Russia a 5-year-old girl, one of two children, was affected. The age at onset was 5–6 months, the phenotype included microcephaly, severe physical, mental and motor developmental delays with spastic paraparesis, accompanied by the absence of walking and speech, and stereotypic movements. Panel MPS “spastic paraplegias” detected a heterozygous novel variant с.3274G > A (p.Val1092Met) in *KIF1A* exon 31. The phenotype is in line with complicated AD SPG30 (or MR9) but the heredity type of the finding was not validated as parents did not present their blood samples. The pathogenicity of the novel variant remains questionable since the mutation is located outside of the *KIF1A* motor domain in contrast to the vast majority of *KIF1A* pathogenic mutations. The diagnosis of SPG30 in this particular case is doubtful and it requires advanced molecular diagnostics with WES to exclude other pathogenic mutations.

## Discussion

While the majority of HSP patients belongs to the SPG4 group and SPG3 incidence rates are occupying the 2nd place in our DNA screening (as SPG4 cases were collected for a longer period), which is corresponding to the world data, the proportion of AD SPG30 has unexpectedly appeared as the 3rd most common SPG form in Russian cohort (8.4%; for the subgroup of AD forms 10.3%). Recently, AD SPG30 cases have been increasingly reported in SPG cohorts from different countries with different incidence rated. Among 192 Italian unrelated HSP patients the MPS panel (targeting genes of 84 SPG and alike disorders) helped to detect 4 different heterozygous *KIF1A* mutations, 2 cases were familiar and 2 sporadic cases (though the total number of DNA-verified cases was not reported) [10]. In another group of 239 Italian families the molecular diagnosis was conducted using MPS panel with 118 target genes (SPG and adjoining disorders). As a result, out of 70 families (29%) with HSP there were 5 families with AD SPG30 (7.1% of all verified cases and 11.0% of AD forms) This makes AD SPG30 form the 2nd most frequent SPG form in the overall incidence statistic, that is being surpassed by only SPG4 [15]. The MPS (93 SPG and ataxias genes) in a Norwegian cohort of 105 patients with HSP and ataxias, that failed to be diagnosed by previous tests, detected two familial AD SPG30 with ‘pure’ cases [14]. In 98 Portugal families the common SPG forms were excluded in 20 cases, and one of those was sporadic complicated SPG30 caused by *KIF1A* mutation that appeared de novo [21]. The panel MPS (SPG and ataxia genes) in 48 HSP unrelated, mostly Dutch patients with earlier performed target DNA testing for several common SPG, detected 2 males (4.2%) with different heterozygous *KIF1A* mutations [22]. Later on, an extensive research of 347 unrelated patients (80% Dutch) by clinical MPS detected 24 (6.9%) SPG30 cases with 20 different *KIF1A* mutations; in 11 families AD inheritance was confirmed, de novo origin of mutations was proven in 7 cases, and inheritance was not established in the remaining 6 patients [16]. The MPS panel screening in a group of 30 Polish HSP families, that have previously shown negative results of several target DNA tests, revealed one familial SPG30 case [23]. MPS in 55 Chinese HSP patients that have previously underwent MLPA screening unraveled 34 cases with SPG mutations with one SPG30 familial case [24]. The referred data show that AD SPG30 contributes to HSP morbidity all over the world, but its proportions are different and some are difficult to be compared. Besides, several groups of patients were pre-selected based on the testing for common SPG forms, in few works a number of all tested families but not of genetically confirmed cases were reported, making the real impact of SPG30 difficult to judge. SPG30 contribution in our cohort (8.4% of all verified cases and 10.3% of AD subgroup) is almost identical to the figures in one of the Italian groups [15] and in Dutch cohort [16] and relatively higher compare to the majority of studies.

Character of mutations in our SPG30 group is in line with reported *KIF1A* mutational spectrum (Table 3). According to the ‘Human Gene Mutation Database’, out of 42 registered *KIF1A* mutations there is only one large deletion detected while the rest represents missense-mutations. The vast majority of *KIF1A* pathogenic mutations as in our research, are located in the motor domain of the gene (up to p.354), but some are not that altogether corresponds to published data [10, 15, 16, 22, 25]. However, in the recent Dutch study a number of mutations located out of the motor domain was unusually high: 9 out of 20 (40.5%), where the rest constitutes of 6 loss-of-function and 3 missense mutations [16]. So, localization of the mutation cannot serve as an absolute criterion of its pathogenicity. Yet we considered the mutation location along with the absence of genealogical confirmation as enough reason to exclude our patient with *KIF1A* variant с.3274G > A (p.Val1092Met) from the SPG30 group. The proportion of 4 novel mutations (Table 2) and of 5 reported earlier (one found twice) is similar to on average observation. We noticed that all novel mutations acquired de novo, while described mutations were mostly found in the subgroup of ‘pure’ familial cases, thus the сase 30–3 is the only one with the ‘pure’ phenotype and a well- known mutation de novo. This mutation p.Ser69Leu was found also in the family 30–4 and was reported before in several families of different ethnicity with pure phenotypes [10, 13, 16, 19, 20], notably, only one patient with this mutation had non-severe additional feature: learning difficulties [20]. Evidently, the mutation may be world-wide spread. Some other mutations were also detected repeatedly, but less often.

**Table 3:** Genetic features of AD SPG30 in 10 Russian families

Family, оrigin	*KIF1A* (NM_004321.6) mutation	Exon	Domain	Familial /sporadic	Origin	GnomAD frequency	LOVD ID	CADD score	Conservativity
30–1 Russian	с.22G > A (Val18Met)	2	Motor	Familial	Maternal	N/D	N/D	27	Conservated
30–2 Russian	с.37С > T (p.Arg13Cys)	2	Motor	Sporadic	De novo	N/D	#0000673886	32	Conservated
30–3, 30–4 Russian, Dargin	с.206C > T (p.Ser69Leu)	4	Motor	Sporadic	De novo	N/D	#0000515392	24.9	Conservated
				Familial	Paternal				
30–5 Tatar	с.220 T > C (p.Tyr74His)	4	Motor	Familial	Paternal	N/D	N/D	27	Conservated
30–6 Russian	с.499C > T (p.Arg167Cys)	5	Motor	Familial	Paternal	N/D	#0000515382	32	Conservated
30–**7** Tatar	c.607A > G (p.Arg203Gln)	6	Motor	Sporadic	De novo	N/D	#0000673887	26.8	Conservated
30–8 Russian	с.761G > A (p.Arg254Gln)	7	Motor	Familial	Maternal	N/D	#0000075855	27.5	Conservated
30–9 Russian- Serb	с.798 + 1G > T	8	Motor	Sporadic	De novo	N/D	#0000673888	34	Conservated
30–10 Russian	c.917A > G (p.Tyr306Cys)	11	Motor	Sporadic	De novo	N/D	#0000673889	26.3	Conservated

*N/D* not detected

Literature data on AD SPG30 clinical characteristics are summarized in Table 4.

**Table 4:** Clinical features of autosomal dominant SPG30

Ref.	IC	Gen der	Age at:		Phenotype P / C	SP	Walking at age of examination	Ataxia	Neuro- pathy	Development delay/mental deficiency	Microcephaly	Epilepsy	Optic atrophy	MRI	Other features
			Exami-nation	SP onset											
[6]	1	F	3.5	N/A	C	*+*	No walking	N/A	N/A	Severe	N/A		–	–	I
[7]	1	F	2	N/A	C	*+*	No walking	N/A	–	Severe	+		–	–	I
	2	M	6	N/A	C	+/−	No walking	N/A	–	Severe	+		+	+	I, II
	3	F	2	N/A	C	*+*	No walking	N/A	–	Severe	–		–	–	I
	4	F	1.5	N/A	C	*+*	No walking	N/A	–	Severe	+		–	–	N/A
	5	M	16	N/A	C	*+*	Aided	+	+	Severe	+		+	**+**	I, II
	6	F	7	N/A	C	*+*	Aided	+	–	Severe	+		–	+	N/A
[8]	1	F	10	Infancy	C	*+*	Unaided	–	–	Moderate	–		–	+	I, II
	2	F	2.5	Infancy	C	*+*	No walking	+	–	Severe	+		+	+	I
	3	F	24	8 mo	C	*+*	Unaided	N/A	+	Mild	–		– §	–	I
	4	M	13	2.5	C	*+*	Unaided	–	+	Mild	–		–	–	N
	5	F	2.5	5 mo	C	*+*	No walking	–	–	Moderate	+		–	+	II
	6	M	4	Infancy	C	*+*	No walking	–	+	Mild	+		+	+	I, II
	7	M	8	20	C	*+*	Aided	–	–	Moderate	–		– §	–	I
	8	F	† 4 y	Congen	C	–	No walking	–	+	Severe	+		+	+	I, II
	9	F	† 2 y	Congen	C	*+*	No walking	N/A	+	Severe	–		N/A	+	I, II
	10	F	10	1 y	C	*+*	Aided	+	+	Mild	–		–	+	I
	11–12 МZ	M	14	1 y	C	*+*	Unaided	–	+	Mild/ Moderate	–		–	–	N
	13	F	15	1.5	C	*+*	Unaided	+	+	Mild	–		–	–	I
	14	F	14	1.5	C	*+*	Aided	–	–	Moderate	–		–	+	N
[9]	1	M	8	5	C	*+*	Unaided	+	–	Mild	–		–	+	I
	2	F	27	1.5	C	*+*	Aided	+	+	Mild	–		–	+	I
	3	F	9	8 mo	C	*+*	Aided	–	+	Severe	–		–	+	I, IV
	4	M	33	7 mo	C	*+*	No walking	+	+	Severe	–		+	+	I, II
	5	F	8	10 mo	C	*+/−*	Unaided	+	–	Mild	–		–	–	I
[10]	1	F	22	< 2	C	*+++*	Aided	–	+	–	–		–	–	N
	2*	M	10	1	C	*++=*	Aided	–	–	Mild	–		–	–	N
	3	M	52	22	P	*++*	Unaided	–	–	–	–		–	–	N
	4*	F	68	63	C	*++*	Unaided	+	–	–	–		–	–	IV
[11]	1	M	14	6 mo	C	*++*	No walking	–	+	Moderate	+		+	+	I, III
	2.	M	6	Congen	C	*++*	No walking	–	+	Moderate	+		–	+	I
[12]	1*	F	7	Congen	C	*+*	Unaided	–	–	Moderate	–		–	+	III, IV
]13]	1*	M	9	2.5	P?	*+*	Unaided	–	–	Borderline	–		– §	–	N
[14]	1*	F	47	12	P	*++*	Aided	–	–	–	–		–	–	N
	2*	M	61	10	P	*++*	Aided	–	–	–	–		–	–	N
[16]	1*	M	56	< 1	P	*+*	Unaided	–	–	–	–		–	–	N/A
	2*	M	14	9	P	*+*	Unaided	–	–	–	–		–	–	N
	3 *	F	17	13	C?	*+*	Unaided	–	–	+/−	–		–	–	N/A
	4 *	M	53	< 10	P	*+*	Aided	–	–	–	–		–	–	N/A
	5	M	38	21	P	*+*	Unaided	–	–	–	–		–	–	N
	6	M	54	18	P	*+*	With aid	–	–	–	–		–	–	N
	7*	M	64	50	C?	*+*	With aid	–	–	+/−	–		–	–	N/A
	8*	M	50	<,10	P	*+*	With aid	–	–	–	–		–	–	N/A
	9*	M	62	57	P	*+*	Unaided	–	–	–	–		–	–	N
	10	M	49	<,10	P	*+*	Unaided	–	–	–	–		–	–	N/A
	11*	F	18	<,10	P	*+*	Unaided	–	–	–	–		–	–	N
	12	F	4	< 1	P	*+*	Aided	–	–	–	–		–	–	III
	13	M	3	1	P	*+*	Unaided	–	–	–	–		–	–	III
	14 *	M	54	<,10	P	*+*	Unaided	–	–	–	–		–	–	N/A
	15*	M	28	~ 20	P	*+*	Unaided	–	–	–	–		–	–	N
	16 *	M	60	~ 30	P	*+*	Unaided	–	–	–	–		–	–	N
	17 *	F	18	1	P	*+*	Unaided	–	–	–	–		–	–	N
	18	F	56	46	P	*+*	Unaided	–	–	–	–		–	–	N
	19	F	51	10	P	*+*	Unaided	–	–	–	–		–	–	N
	20*	F	66	<,10	P	*+*	Unaided	–	–	–	–		–	–	N/A
	21	M	12	2	P	*+*	Unaided	–	–	–	–		–	–	N/A
	22*	M	22	2	P	*+*	Unaided	–	–	–	–		–	–	N/A
	23	M	20	< 5	P	*+*	Unaided	–	–	–	–		–	–	N
	24	M	6/5	1	C?	*+*	Unaided	–	–	Mild	–		–	–	N
[19]	1*	M	61	3	P	*++*	No walking	–	–	–	–		–	–	N
[20]	1	M	6 mo	Congen	C	*+*	No walking	–	+	Mild	N/A		+	+	I, III
	2	F	21 mo	Congen	C	*+*	No walking	–	+	Mild	N/A		+	–	I, II, III
	3	F	11	1	C	*+*	Aided	+	N/A	N/A	N/A		+	+	I, IV
	4	F	9	6 mo	C	*+*	Aided	–	–	–	N/A		+	–	I, III
	5A-B MZ	F	21	< 1	C	–	Aided	–	+	Mild	N/A		+	+	I, III, IV
	6	M	11	Antenat	C	*+*	Unaided	+	–	–	N/A		–	+	I, III
	7	M	12	9 mo	C	*+/−*	Unaided	–	+	Mild	N/A		+	–	III
	8*	F	10	1.5	C	*+*	Unaided.	–	–	–	N/A		–	–	N
	9	M	11	< 1	C	*+/−*	N/A	–	+	Mild	N/A		–	–	SAC
	10	F	10	2nd year	C	*+*	Unaided	–	+	Mild	N/A		–	–	N
[21]	1	M	13	1	C	*+* mild	Unaided	–	+	–	–		–	–	N
[22]	1*	M	N/A	< 3 mo	P	*++*	N/A	–	–	–	–		–	–	N
	2*	M	N/A	< 20	P	*++*	N/A	–	–	–	–		–	–	N
[23]	1*	F	N/A	Child- hood	C	*++*	N/A	N/A	N/A	Mild	N/A		N/A	–	N/A
[24]	1*	M	20	1	C	***+***	Unaided	+	+	Mild	–		–	–	I
[25]	1	M	18	Infan	C	*+*	Aided	–	–	Severe	+		+	–	N
	2	F	10	9 mo	C	*+*	No walking	+	–	Severe	–		–	N/A	I, II
	3	F	14	4 mo	C	*+*	Aided	–	+	Moderate	–		–	+	N
	4	F	15	11 mo	C	*+*	Aided	–	–	Moderate	–		–	–	I
[26]	1	M	20	18	P?	*++*	Unaided	–	+/−	–	–		–	–	N/A
[27]	1	M	17	12	C	*++*	Unaided	–	–	–	–		+	+	I, III
[28]	1	M	8	8 mo	C	*++*	No walking	N/A	–	Severe	+		+	+	I, III, HH
[29]	1	F	47	12	C	*+*	Unaided	–	+	Moderate	–		+	–	I, II
[30]	1	M	4	N/A	C	*+*	No walking	N/A	+	Severe	–		+	+	I, II, III
[31]	1	M	19	1.5	C	*++*	Unaided	+/−	+	Mild	+		–	+	I
[32]	1	M	**7**	6 mo	C	*+*	Unaided	+/−	+	Mild	–		–	–	I

In familial cases, only index cases are included. AR SPG30 and non-SPG KIF1A-related phenotypes are not included

Legend: *IC* index case, *SP* spastic paraparesis, *P* pure SP, *C* complicated SP, * familial case, † death, *N* normal, + present, − absent, *N/A* not available, § electroencephalographic epileptiform activity, *MZ* monozygous twins, *ADD* attention deficit disorder, *ASD* autistic spectrum disorder; MRI signs: I – cerebellar atrophy, II – cerebral atrophy, III – hypogenesia of corpus callosum; *WM* white matter lesion, *SAC* small arachnoidal cysts, *HH* hypoplasia of hypophysis

‘Pure’ AD SPG30 have typical, well-known features of spastic paraparesis. Early manifestation and slow course, like in the most of our cases, are common though age of onset may vary: from 1 year up to the 7th decade in 7 Italian patients [10], from congenital up to 54 years in Dutch groups [16, 22]. Clinically ‘pure’ AD SPG30 are indistinguishable from well-known common SPG3 or early forms of SPG4. Interfamilal variability of the clinical picture in our group includes the age of onset (case 30–6) and incomplete penetrance (mutation carriers with subclinical signs in cases 30–1 and 30–5). Incomplete penetrance is not common in AD SPG30, but few cases were reported, for example in a family with an early onset and slow course one of carriers had only hyperreflexia in 31 year [14].

Complicated familial cases have been already reported beyond our study. In some families, patients uniformly developed additional non-classical features, identical [23] or differing [12], in other cases ‘pure’ and complicated phenotypes were combined [10, 13]. Altogether this points to the absence of strict ‘genotype-phenotype’ correlation in SPG30. The most common additional symptom in complicated cases is cognitive deficiency. ASD and attention deficit disorder with hyperactivity (ADHD) were also reported in several SPG30 cases. In the Finnish family ADHD with learning difficulties in an affected boy was considered to be independent of clinical phenotype since his father had ‘pure’ SPG30 [13]. However newly reported cases of ASD and/or ADHD in SPG30 [25–27] permit to regard them as possible parts of SPG30 phenotypes. Our case 30–9 may be in line with the idea though special examination of the patient could not be performed.

The vast majority of cases that have developed due to de novo mutations are complicated, e.g. with various additional signs presented in different combinations, that are characterized by variable manifestation age and by different severity, like in our cases 30–2 and 30–7. Our ‘pure’ case 30–3 with mutation de novo is most likely a rare exception. The main, practically obligatory additional sign in complicated SPG30 is mental deficiency ranging from severe (more often) to mild grades. Common signs are microcephaly, epilepsy, optic atrophy, ataxia, axonal neuropathy, dystonia etc. MRI abnormalities (cerebral and/or cerebellar atrophy, hypogenesia/ thinning of corpus callosum, white matter lesion), epilepsy, optic atrophy, blindness of central origin, axonal neuropathy, axial hypotony, athetosis, dystonia, etc. Scoliosis and contractures are common secondary skeletal lesions. Short stature was one of additional signs in two unrelated Japan patients [28, 29]. Almost each complicated SPG30 case, sporadic or familial, has individual set of additional symptoms and/or the disease course. Our 4 sporadic cases may add to this variability.

mutations acquired de novo [6, 9, 11, 24, 26–33] several groups of sporadic cases were reported: 4 Dutch patients [25], 6 patients age 1.5–16 years from USA [7], 5 cases among 62 unrelated patients with cerebral atrophy of unestablished nature [9]. In addition, one of the first cohorts with de novo mutations in *KIF1A* was a representative international group (Canada, USA, Netherlands and Finland): 14 patients (monozygotic twin pair among them) age 2.5–24 years, all had de novo mutations in *KIF1A* [8]. Recently, another international group was described, where 9 out of 10 patients (one of them also was monozygotic twin pair) had *KIF1A* mutations acquired de novo and only one was AD familial case [20]. Altogether sporadic SPG30 cases contribute significantly into AD SPG30 world pool.

Other *KIF1A*-related phenotypes with AD inheritance exist. Thus, several cases with phenotype of PEHO syndrome (Progressive encephalopathy with edema, hypsarrhythmia, and optic atrophy) and with mutated *KIF1A* were reported [20, 34, 35] (where ‘classic’ PEHO is severe AR disorder produced by *ZNHIT3* mutations). Another case with a novel *KIF1A* mutation acquired de novo is describing a girl with typical Rett syndrome that was previously negatively tested for mutations in ‘Rett genes’ *MeCP2*, *CDKL5*, and *FOXG1* [36]. Novel *KIF1A* mutation was the only significant molecular finding in a Chinese family with AD epilepsy (6 patients in 3 generations and diabetes in some of them but no other signs) [37]. *KIF1A* mutation was detected in one of 92 patients with infantile spasms of unestablished etiology [33]. Our case 30–10 with minimal spasticity, moderate ataxia, benign course with gradual improvement and non-severe congenital cataract that has not been reported previously may present a new variant of AD *KIF1A*-related phenotype. Yet SPG phenotypes are the most common within *KIF1A-*driven pathologies.

Apart from AR SPG30, another *KIF1A*-related disorder with AR inheritance was described in 2011, namely AR sensory and autonomic neuropathy type II (HSAN II, OMIM#614213) was described in 4 families: consanguineous Turkish and Afghan families with 3 patients in each and common mutation; and two Belgian cases: a pair of monozygotic twins and a single compound heterozygous patient [38]. The disease had the onset from congenital to early adolescence, and severe course. No new cases have been described since.

## Conclusion

AD SPG30 caused by various heterozygous *KIF1A* mutations is one of the most common HSP in Russian population: 10 SPG30 families amounted for 8.4% from the total 118 cases, that have been molecularly diagnosed by MPS panel -‘spastic paraplegias’. Four newly-detected *KIF1A* mutations contribute to the gene mutational spectrum. The mutation p.Ser69Leu in the exon 3 was found in two families of different ethnicity and it have been previously reported in a number of cases. Altogether, that may suggest that this mutation is relatively common. Six ‘pure’ cases (5 familial, one with the mutation de novo) were phenotypicaly similar (early onset and slow course prevailed), 2 of those mutations demonstrated incomplete penetrance. Four sporadic complicated cases were clinically heterogeneous. Two patients had severe MR, one child never walked. Female patient with a borderline intelligence had atypical movement disorders: prevailing ataxia with minimal spasticity plus cataract, a feature that have not been reported earlier. The Russian group confirms SPG30 frequency and pronounced clinical variability.

## Data Availability

The authors declare that all the raw data are contained within the Table 1 of the manuscript.

## Abbreviations

SP30: Spastic paraplegia type 30
AR: Autosomal recessive
AD: Autosomal dominant
HSP: Hereditary spastic paraplegias
SPG: Spastic Paraplegia Gene
MPS: Massive parallel sequencing
MR: Mental retardation
IC: Index cases
WES: Whole-exome sequencing
MLPA: Multiplex Ligation-dependent Probe Amplification
MRI: Magneticresonance imaging
EEG: Electroencephalography
ASD: Autistic spectrum disorder
OS: Oculus sinister

## References

[1] Gabrych DR, Lau VZ, Niwa S, Silverman MA (2019). Going too far is the same as falling short: kinesin-3 family members in hereditary spastic paraplegia. Front Cell Neurosci.

[2] Klebe S, Azzedine H, Durr A, Bastien P, Bouslam N, Elleuch N, Forlani S, Charon C, Koenig M, Melki J (2006). Autosomal recessive spastic paraplegia (SPG30) with mild ataxia and sensory neuropathy maps to chromosome 2q37.3. Brain..

[3] Erlich Y, Edvardson S, Hodges E, Zenvirt S, Thekkat P, Shaag A, Dor T, Hannon GJ, Elpeleg O (2011). Exome sequencing and disease-network analysis of a single family implicate a mutation in KIF1A in hereditary spastic paraparesis. Genome Res.

[4] Klebe S, Lossos A, Azzedine H, Mundwiller E, Sheffer R, Gaussen M, Marelli C, Nawara M, Carpentier W, Meyer V (2012). KIF1A missense mutations in SPG30, an autosomal recessive spastic paraplegia: distinct phenotypes according to the nature of the mutations. Eur J Hum Genet.

[5] Krenn M, Zulehner G, Hotzy C, Rath J, Stogmann E, Wagner M, Haack TB, Strom TM, Zimprich A, Zimprich F (2017). Hereditary spastic paraplegia caused by compound heterozygous mutations outside the motor domain of the KIF1A gene. Eur J Neurol.

[6] Hamdan FF, Gauthier J, Araki Y, Lin DT, Yoshizawa Y, Higashi K, Park AR, Spiegelman D, Dobrzeniecka S, Piton A (2011). Excess of de novo deleterious mutations in genes associated with glutamatergic systems in nonsyndromic intellectual disability. Am J Hum Genet.

[7] Esmaeeli Nieh S, Madou MR, Sirajuddin M, Fregeau B, McKnight D, Lexa K, Strober J, Spaeth C, Hallinan BE, Smaoui N (2015). De novo mutations in KIF1A cause progressive encephalopathy and brain atrophy. Ann Clin Transl Neurol.

[8] Lee JR, Srour M, Kim D, Hamdan FF, Lim SH, Brunel-Guitton C, Décarie JC, Rossignol E, Mitchell GA, Schreiber A (2015). De novo mutations in the motor domain of KIF1A cause cognitive impairment, spastic paraparesis, axonal neuropathy, and cerebellar atrophy. Hum Mutat.

[9] Ohba C, Haginoya K, Osaka H, Kubota K, Ishiyama A, Hiraide T, Komaki H, Sasaki M, Miyatake S, Nakashima M (2015). De novo KIF1A mutations cause intellectual deficit, cerebellar atrophy, lower limb spasticity and visual disturbance. J Hum Genet.

[10] Citterio A, Arnoldi A, Panzeri E, Merlini L, D'Angelo MG, Musumeci O, Toscano A, Bondi A, Martinuzzi A, Bresolin N, Bassi MT (2015). Variants in KIF1A gene in dominant and sporadic forms of hereditary spastic paraparesis. J Neurol.

[11] Hotchkiss L, Donkervoort S, Leach ME, Mohassel P, Bharucha-Goebel DX, Bradley N, Nguyen D, Hu Y, Gurgel-Giannetti J, Bönnemann CG (2016). Novel de novo mutations in KIF1A as a cause of hereditary spastic paraplegia with progressive central nervous system involvement. J Child Neurol.

[12] Cheon CK, Lim SH, Kim YM, Kim D, Lee NY, Yoon TS, Kim NS, Kim E, Lee JR (2017). Autosomal dominant transmission of complicated hereditary spastic paraplegia due to a dominant negative mutation of KIF1A, SPG30 gene. Sci Rep.

[13] Ylikallio E, Kim D, Isohanni P, Auranen M, Kim E, Lönnqvist T, Tyynismaa H (2015). Dominant transmission of de novo KIF1A motor domain variant underlying pure spastic paraplegia. Eur J Hum Genet.

[14] Iqbal Z, Rydning SL, Wedding IM, Koht J, Pihlstrøm L, Rengmark AH, Henriksen SP, Tallaksen CM, Toft M (2017). Targeted high throughput sequencing in hereditary ataxia and spastic paraplegia. PLoS One.

[15] D'Amore A, Tessa A, Casali C, Dotti MT, Filla A, Silvestri G, Antenora A, Astrea G, Barghigiani M, Battini R (2018). Next generation molecular diagnosis of hereditary spastic paraplegias: an Italian cross-sectional study. Front Neurol.

[16] Pennings M, Schouten MI, van Gaalen J, Meijer RPP, de Bot ST, Kriek M, Saris CGJ, van den Berg LH, van Es MA, Zuidgeest DMH (2020). KIF1A variants are a frequent cause of autosomal dominant hereditary spastic paraplegia. Eur J Hum Genet.

[17] Richards S, Aziz N, Bale S, Bick D, Das S, Gastier-Foster J, Grody WW, Hegde M, Lyon E, Spector E (2015). Standards and guidelines for the interpretation of sequence variants: a joint consensus recommendation of the American College of Medical Genetics and Genomics and the Association for Molecular Pathology. Genet Med.

[18] Ryzhkova OP, Kardymon OL, Prohorchuk EB, Konovalov FA, Maslennikov AB, Stepanov VA, Afanasyev AA, Zaklyazminskaya EV, Rebrikov DV, Savostyanov KV (2019). Guidelines for the interpretation of data on human DNA sequencing obtained by methods of massive parallel sequencing (MPS) (Ed.2018, version 2). Med Genet.

[19] Roda RH, Schindler AB, Blackstone C (2017). Multigeneration family with dominant SPG30 hereditary spastic paraplegia. Ann Clin Transl Neurol.

[20] Nemani T, Steel D, Kaliakatsos M, DeVile C, Ververi A, Scott R, Getov S, Sudhakar S, Male A, Mankad K. Genomics England Research Consortium et al. KIF1A-related disorders in children: a wide spectrum of central and peripheral nervous system involvement. J Peripher Nerv Syst. 2020. 10.1111/jns.12368. 10.1111/jns.12368 32096284

[21] Morais S, Raymond L, Mairey M, Coutinho P, Brandão E, Ribeiro P, Loureiro J, Sequeiros J, Brice A, Alonso I, Stevanin G (2017). Massive sequencing of 70 genes reveals a myriad of missing genes or mechanisms to be uncovered in hereditary spastic paraplegias. Eur J Hum Genet.

[22] van de Warrenburg BP, Schouten MI, de Bot ST, Vermeer S, Meijer R, Pennings M, Gilissen C, Willemsen MA, Scheffer H, Kamsteeg EJ (2016). Clinical exome sequencing for cerebellar ataxia and spastic paraplegia uncovers novel gene-disease associations and unanticipated rare disorders. Eur J Hum Genet.

[23] Elert-Dobkowska E, Stepniak I, Krysa W, Ziora-Jakutowicz K, Rakowicz M, Sobanska A, Pilch J, Antczak-Marach D, Zaremba J, Sulek A (2019). Next- generation sequencing study reveals the broader variant spectrum of hereditary spastic paraplegia and related phenotypes. Neurogenetics..

[24] Lu C, Li LX, Dong HL, Wei Q, Liu ZJ, Ni W, Gitler AD, Wu ZY (2018). Targeted next-generation sequencing improves diagnosis of hereditary spastic paraplegia in Chinese patients. J Mol Med (Berl).

[25] Van Beusichem AE, Nicolai J, Verhoeven J, Speth L, Coenen M, Willemsen MA, Kamsteeg EJ, Stumpel C, Vermeulen RJ (2020). Mobility сharacteristics of сhildren with spastic paraplegia due to a mutation in the KIF1A gene. Neuropediatrics..

[26] Tomaselli PJ, Rossor AM, Horga A, Laura M, Blake JC, Houlden H, Reilly MM (2017). A de novo dominant mutation in KIF1A associated with axonal neuropathy, spasticity and autism spectrum disorder. J Peripher Nerv Syst.

[27] Kurihara M, Ishiura H, Bannai T, Mitsui J, Yoshimura J, Morishita S, Hayashi T, Shimizu J, Toda T, Tsuji S (2020). A novel de novo KIF1A mutation in a patient with autism, hyperactivity, epilepsy, sensory disturbance, and spastic paraplegia. Intern Med.

[28] Okamoto N, Miya F, Tsunoda T, Yanagihara K, Kato M, Saitoh S, Yamasaki M, Kanemura Y, Kosaki K (2014). KIF1A mutation in patient with progressive neurodegeneration. J Hum Genet.

[29] Yoshikawa K, Kuwahara M, Saigoh K, Ishiura H, Yamagishi Y, Hamano Y, Samukawa M, Suzuki H, Hirano M, Mitsui Y (2018). The novel de novo mutation of KIF1A gene as the cause for spastic paraplegia 30 in a Japanese case. Neurol Sci.

[30] Raffa L, Matton MP, Michaud J, Rossignol E, Decarie JC, Ospina LH (2017). Optic nerve hypoplasia in a patient with a de novo KIF1A heterozygous mutation. Can J Ophthalmol.

[31] Spagnoli C, Rizzi S, Salerno GG, Frattini D, Fusco C (2019). Long-term follow-up until early adulthood in autosomal dominant, complex SPG30 with a novel KIF1A variant: a case report. Ital J Pediatr.

[32] Urtiaga Valle S, Fournier Gil B, Ramiro León MS, Martínez MB. Usefulness of exome sequencing in the study of spastic paraparesis and cerebellar atrophy: De novo mutation of the KIF1A gene, a new hope in prognosis. Neurologia. 2019;S0213-4853(18)30197-X. 10.1016/j.nrl.2018.07.001. 10.1016/j.nrl.2018.07.001 30862385

[33] Muir AM, Myers CT, Nguyen NT, Saykally J, Craiu D, De Jonghe P, Helbig I, Hoffman-Zacharska D, Guerrini R, Lehesjoki AE (2019). Genetic heterogeneity in infantile spasms. Epilepsy Res.

[34] Langlois S, Tarailo-Graovac M, Sayson B, Drögemöller B, Swenerton A, Ross CJ, Wasserman WW, van Karnebeek CD (2016). De novo dominant variants affecting the motor domain of KIF1A are a cause of PEHO syndrome. Eur J Hum Genet.

[35] Samanta D, Gokden M (2019). PEHO syndrome: KIF1A mutation and decreased activity of mitochondrial respiratory chain complex. J Clin Neurosci.

[36] Wang J, Zhang Q, Chen Y, Yu S, Wu X, Bao X (2019). Rett and Rett-like syndrome: expanding the genetic spectrum to KIF1A and GRIN1 gene. Mol Genet Genomic Med.

[37] Guo Y, Chen Y, Yang M, Xu X, Lin Z, Ma J, Chen H, Hu Y, Ma Y, Wang X, Tian X (2020). A rare KIF1A missense mutation enhances synaptic function and increases seizure activity. Front Genet.

[38] Rivière JB, Ramalingam S, Lavastre V, Shekarabi M, Holbert S, Lafontaine J, Srour M, Merner N, Rochefort D, Hince P (2011). KIF1A, an axonal transporter of synaptic vesicles, is mutated in hereditary sensory and autonomic neuropathy type 2. Am J Hum Genet.

